# Uncommon Carotid Artery Stenting Complications: A Series by Images

**DOI:** 10.3390/jpm14030250

**Published:** 2024-02-26

**Authors:** Giuseppe Vadalà, Vincenzo Sucato, Francesco Costa, Fausto Castriota, Roberto Nerla, Giuseppe Roscitano, Antonio Giovanni Versace, Alfredo Ruggero Galassi, Antonio Micari

**Affiliations:** 1 Division of Cardiology, University Hospital Paolo Giaccone, 90100 Palermo, Italy; 2 Department of Health Promotion, Mother and Child Care, Internal Medicine and Medical Specialties (ProMISE), University of Palermo, 90146 Palermo, Italy; 3 Interventional Cardiology Department, Policlinic G. Martino, University of Messina, 98122 Messina, Italy; 4 Cardio-Vascular Department, Maria Cecilia Hospital, GVM Care and Research, 48100 Ravenna, Italy; 5 Department of General Surgery and Medical Specialities, University of Catania, 95123 Catania, Italy; 6 Internal Medicine Department, AO Papardo, 98100 Messina, Italy

**Keywords:** carotid artery stenting (CAS) complications, acute carotid stent thrombosis, cerebral embolic protection device failure, plaque prolapse after carotid stenting, cerebral hyper perfusion Syndrome (CHS), radial access site complications

## Abstract

Aims: To describe through emblematic images rare but clinically relevant carotid artery stenting complications that occurred at two high-volume centres for carotid artery stenting (CAS). Background: CAS is an alternative to carotid endarterectomy (CEA) for the treatment of carotid artery stenosis in patients judged to be at high risk for CEA. CAS complications range between 1 and 9% and are higher in older patients complaining of neurological symptoms at the time of presentation. Besides periprocedural or early-after-procedure stroke, which remains the true Achilles’ heel of CAS, other dramatic complications might compromise the clinical outcomes of this procedure. Methods: Five infrequent complications, out of more than 1000 CAS performed in the years 2016–2021, have been described. Results: Among CAS complications, acute carotid stent thrombosis, rescue retrieval of a disconnected distal cerebral embolic protection device, plaque prolapse after carotid stenting, cerebral hyperperfusion syndrome (CHS), and radial artery long sheath entrapment requiring surgical intervention were found to account for 0.3% of the total number of procedures performed by operators with high CAS volume. Conclusions: Unusual CAS complications may infrequently occur, even in hands of expert operators. To know how to deal with such complications might help interventionalists to improve CAS performance.

## 1. Introduction

Carotid artery stenting (CAS) is an alternative to carotid endarterectomy (CEA) for the treatment of carotid artery stenosis in patients judged to be at high surgical risk [1]. Many studies comparing CEA to CAS suggested that the first is more frequently complicated by periprocedural myocardial infarction while the latter is more frequently complicated by stroke [2]. Periprocedural CAS complications range roughly between 1 and 9%, being higher in older patients with prior neurological symptoms [3]. Despite the enormous advancements in technology and procedural techniques, such as the availability of dedicated stents, cerebral embolic protection devices (CEPDs), calcific plaque-modifying systems [4,5], or the use of radial access, periprocedural stroke still represents the true Achilles’ heel of CAS. Aortic arch and carotid artery plaque embolization during carotid vessel negotiation and plaque prolapse after stent deployment are the most common causes of periprocedural stroke; conversely, acute stent thrombosis and hyperperfusion syndrome are very rare complications [3]. In addition to neurological complications, vascular-access-site-related complications might be clinically relevant as well. Although, in the last few years, TRA has become an alternative access route for even peripheral interventions, most CAS procedures are still performed through trans-femoral access (TFA) [6]. Allowing for the fact that an extensive adoption of TRA for percutaneous coronary interventions (PCIs) has dramatically reduced the rates of bleeding complications, mortality, and hospital stay for TFA, this aspect could be clinically relevant for CAS procedures as well [7,8]. However, a systematic description of specific TRA complications in carotid interventions is still lacking in the literature. The aim of the present study is to describe, through emblematic images, some extremely infrequent but clinically relevant CAS complications which occurred at two referral hospitals for CAS procedures in the hands of highly skilled operators. These complications might potentially lead to procedural stroke, threaten the vascular access site, and prolong hospital stay definitively. To share this knowledge might help readers to understand the underlying mechanisms and possible solutions for these true nightmares for interventionalists involved in CAS procedures.

## 2. Materials and Methods

One thousand and twelve CAS procedures performed at two high-volume referral centres for peripheral interventions from 2015 to 2021 were retrospectively evaluated. Written informed consent was obtained from all subjects involved in the study and the local ethical committee approved the manuscript’s production.

Clinical, anatomic, and procedural characteristics were derived from patients’ electronic clinical files. Procedural angiograms, duplex scan images, and intravascular images were reviewed by two interventionalists to define lesion characteristics, technical success, and complications. In case of disagreement, a third operator reviewed the data. All the patients were scheduled for a CAS procedure if they were judged at high risk for CEA, if they had a life expectancy > 5 years, and in case of stenosis > 50% if symptomatic or >80% if asymptomatic. Among all procedural complications, those that are very uncommon (≤0.1%) represent the main object of our study.

Concomitant therapy: All patients were on dual-antiplatelet therapy before CAS procedure (acetyl salicylic acid 100 mg plus clopidogrel 75 mg). After the procedure, clopidogrel therapy was continued for at least 1 month, while aspirin was continued indefinitely. For intra-procedural anticoagulation, unfractionated heparin (70–100 IU/kg) was administered to maintain an activated clotting time > 250 s.

Definitions. Technical success was defined as angiographic success (final residual stenosis < 30% by visual estimation). Procedural success was defined as technical success with no in-hospital major adverse cardiac and cerebrovascular events (MACCEs), a composite endpoint including death, myocardial infarction (MI), stroke, and recurrent symptoms requiring repeat target vessel revascularization. Neurological complications were classified as one of the following: minor stroke, defined as a new neurological deficit that either was resolved completely within 30 days or determined an increase in the National Institute of Health Stroke scale (NIHSS) score of ≤3; major stroke, defined as a new neurological deficit that persisted for >30 days and increased the NIH Stroke Scale score by ≥4; and amaurosis fugax, defined as a temporary monocular loss of vision [9]. Major bleeding was defined as type 3 or higher bleeding according to the Bleeding Academic Research Consortium (BARC) criteria. Major access site complication was defined as major bleeding at the site of vascular access or hematomas ≥10 cm in diameter leading to a prolonged hospital stay [10].

## 3. Results

Clinical and procedural characteristics are described in detail in Table 1. The main findings of the study are highlighted in the Central Illustration. Technical success was achieved in 1007 of 1012 procedures (99.5%); the remaining 5 procedures were unsuccessful due to a final residual stenosis > 50% in 4 cases (0.4%) and due to unsuccessful stent deployment in 1 case (0.1%). Procedural success was achieved in 943 procedures out of 1007 (93.7%); procedural and in-hospital MACCEs occurred in 64 procedures (6.3%). All the details are reported in Table 2. Vascular complications occurred in fifty-four procedures (5.3%); in four cases, a surgical intervention at the vascular site was needed (0.4%), and in eight cases (0.8%), a blood transfusion was required. Finally, the five very uncommon complications identified were the following: (1) one case of acute stent thrombosis; (2) one case of hyperperfusion syndrome; (3) one case of embolic protection device embolization; (4) one case of plaque prolapse; and (5) one case of 90 cm long radial sheath entrapment that required surgical removal. All cases and their outcomes are described hereafter.

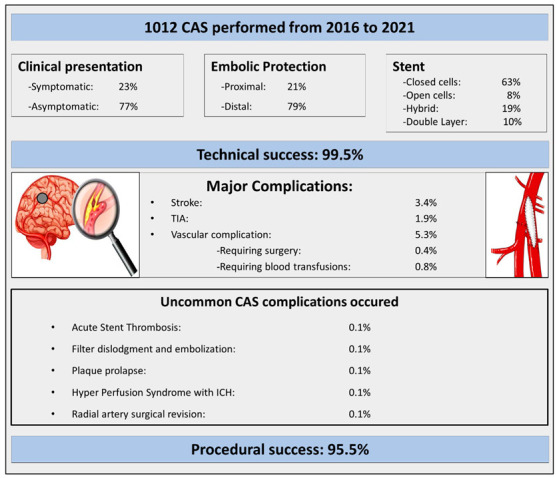



**Central Illustration.** Uncommon CAS complications that occurred among one thousand consecutive CAS procedures performed by highly skilled operators at two referral centres for CAS. CAS = carotid artery stenting; ICH = intracranial haemorrhage; TIA = transient ischemic attack.

**(1)**  **Acute Carotid Stent thrombosis:**

Acute carotid stent thrombosis (ACST) is a rare and dreadful complication previously described in the literature. It occurs in 0.05% to 0.8% of procedures, usually within 1–2 h after stenting, and requires a prompt treatment to avoid potential catastrophic neurological sequelae [11]. Antiplatelet medication noncompliance or discontinuation, antiplatelet medication resistance, overlapping stent placement, or intrinsic prothrombotic disorders have been described as possible predisposing risk factors. In addition, stent edge dissection, atheroma disruption, embolic protection device failure, or ICA kinking after stent placement may favour stent occlusion as well [12]. However, a systematic approach to ACST is lacking and different treatment options have been described: medical therapy (both anticoagulant and or thrombolytic agents), endovascular treatment, surgical stent explant, or a combination of these approaches [13].

**Brief Case description:**

**Figure 1:**
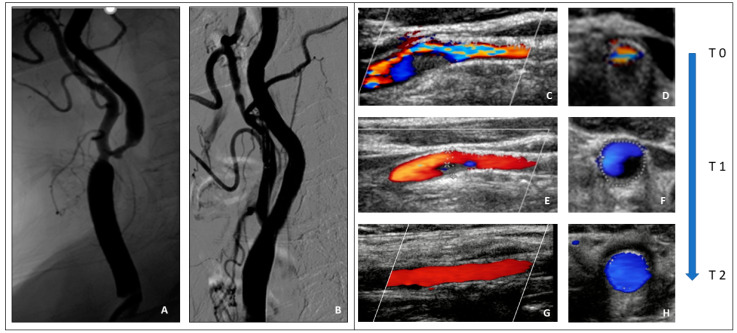
(**A**,**B**) Right carotid artery digital subtraction angiography (DSA). (**A**). Ulcerated severe ICA stenosis. (**B**). Result after carotid stenting (Xact 8–10/40 mm). (**C**–**H**) Carotid duplex scan, in long and short axis views. (**C**,**D**)—Time 0. Thrombotic formation at the stent distal edge. (**E**,**F**)—Time 1. Partial reduction of thrombotic burden. (**G**,**H**)—Time 2. Complete lysis of the thrombus.

A 78-year-old lady, who was complaining of a recent transient ischemic attack (TIA) characterized by dysarthria and right amaurosis fugax and was judged to be at high risk for CEA due to a concomitant severe coronary artery disease, underwent right carotid stenting. The procedure was performed using a proximal embolic protection device (MOMA, Medtronic, Inc., Minneapolis, MN, USA). After lesion pre-dilatation with an undersized semi-compliant balloon (2.5/20 mm), an 8–10/30 mm Xact stent (Abbott, Illinois, USA) was implanted, followed by a 5.5/20 mm semi-compliant balloon post dilatation. The angiographic result was satisfactory. Before arterial sheath removal, the activated clotting time was 256 s; 3 mg intravenous protamine was used to partially revert the periprocedural unfractionated heparin (UFH). After that, the patient complained of dysarthria and left hemiplegia. A cerebral CT excluded haemorrhagic or acute ischemic lesions, while the carotid duplex scan showed a subtotal thrombotic occlusion of the internal carotid artery close to the distal edge of the stent. A full dose of UFH was administered and within the next two hours a progressive improvement of the neurological symptoms was observed. Moreover, a series of carotid duplex scans demonstrated a progressive and complete spontaneous lysis of the thrombus. The angio-CT scan excluded any possible “stent-related” reasons for acute thrombosis such as vessel dissection, stent under expansion, or plaque prolapse. The assumed pathophysiological cause of stent thrombosis was a thrombotic disorder related to an unknown, at the time of procedure, antiphospholipid syndrome and the concomitant administration of protamine before sheath removal (Figure 1).

**(2)**  **Rescue retrieval of a disconnected distal cerebral embolic protection device**

According to the latest guidelines and state-of-the-art papers, the use of embolic protection devices (EPDs) for carotid artery stenting to reduce the risk of procedural cerebral embolization is recommended [1,3]. However, possible EPD-related complications exist, like locking between the stent-delivering catheter and the EPD, separation of the membranous component from the device, inability to cross the stent with the retrieval sheath, retained EPD, and fractured guidewire. The treatment options reported are endovascular rescue or carotid endarterectomy [14,15].

**Brief Case description:**

**Figure 2:**
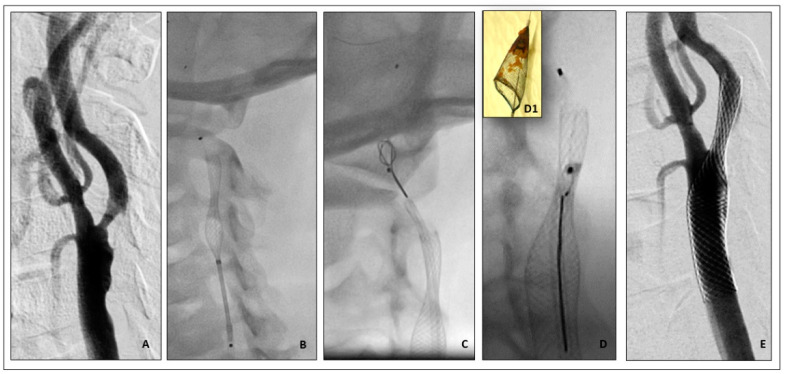
(**A**) Digital subtraction angiography (DSA) shows severe internal carotid artery (ICA) stenosis. (**B**) Deployment of 7.0/40 mm Carotid WallStent (Boston Scientific, USA). Embolic protection with Spider Filter (Medtronic, USA) in distal ICA. (**C**,**D**) Filter basket snaring and retrieval. (**D1**) Filter basket full of plaque debris. (**E**) Final result after carotid stenting.

A 73-year-old man with previous percutaneous coronary intervention (PCI) and peripheral lower limb angioplasty was scheduled for right CAS after he complained of a minor stroke characterized by left arm hyposthenia and multiple right cortico-cerebral ischemic lesions on cerebral CT scan. The procedure was performed through a 6 F Internal Mammary (IM) guiding catheter advanced into the distal right CCA. The stenosis was crossed with a 0.014” Choice extra support wire (Boston Scientific, Marlborough, MA, USA) and a 6 mm Spider-FX Filter (Medtronic, Inc. USA) was deployed over this wire up to the distal portion of the ICA. After stenting by a 7.0 × 40 mm Carotid Wall Stent (Boston Scientific, Marlborough, MA, USA), a filter disconnection was identified a few millimetres away from the transition between the filter’s basket and the filter wire. The IM guiding catheter was advanced through the stent close to the filter and it was recaptured using a 4 mm snare device. During the attempt to recapture the filter, the patient complained of aphasia which resolved within 5 h. Despite the fact that a stent was not performed post dilatation, the periprocedural duplex scan showed a satisfactory stent expansion as the minimal lumen stent diameter was 4.2 mm, and the spectral Doppler peak systolic velocity was normal. The cerebral CT scan performed on the day after the procedure did not show any cerebral acute ischemic injury (Figure 2).

**(3)**  **Plaque prolapse after carotid stenting**

Plaque prolapse through stent struts after carotid artery stenting might lead to ischemic stroke at the time of stenting and in the early post-procedural phase. Thanks to the adoption of a multi-imaging approach in peripheral interventions, this condition can be frequently observed using intravascular optical coherence tomography (OCT) [16]. It was recently demonstrated that plaque protrusion with attenuation on OCT is an independent risk factor for new periprocedural brain lesions detected by MRI after carotid stenting, especially in cases of vulnerable plaque stenting [17]. Nowadays, vulnerable carotid plaques can be accurately identified by high-resolution magnetic resonance imaging. Indeed, the presence of plaque surface ulceration lipid-rich necrotic cores (LRNC, >40% of plaque volume), thin fibrous caps (<165 µm), intraplaque haemorrhage (IPH), and positive vascular remodelling strongly suggest the plaque might be prone to rupture [18]. Self-expanding covered stents might potentially reduce the risk of cerebral micro embolism during and after carotid stenting, but a very early trial was prematurely stopped because of inacceptable in-stent restenosis rates [19]. Conversely, the use of the newest carotid stents, such as double-layer mash stents, has significantly reduced plaque prolapse rates and new periprocedural brain lesions detected by MRI [20].

**Brief case description**

**Figure 3:**
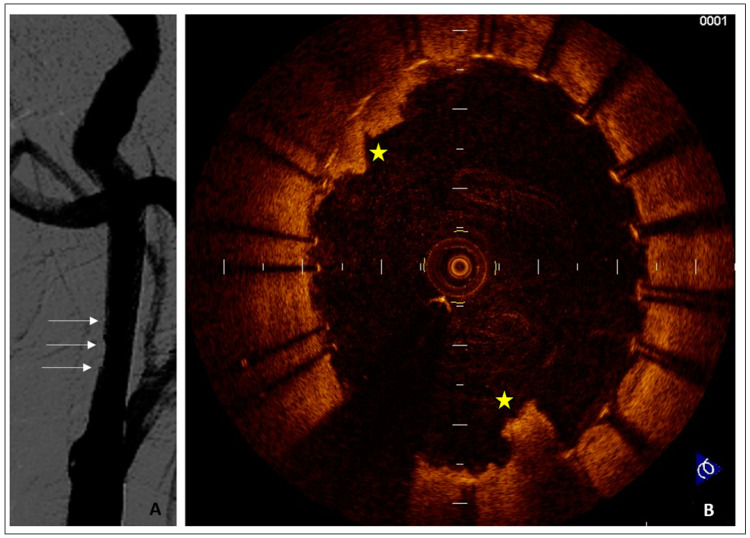
(**A**) Angiographic result after carotid artery stenting with a Carotid WallStent 7–40 mm (Boston, USA). The yellow arrow points to a modest plaque prolapse. (**B**) OCT image showing plaque protrusion beyond stent struts between 8 and 11 and at 5 o’clock (yellow stars).

A 71-year-old man, complaining of minor stroke, with a previous neck X-ray irradiation of a laryngeal carcinoma, was scheduled for left carotid stenting. The procedural setting was as follows: distal cerebral protection by Filter EZ (Boston Scientific, USA), direct stenting with a Carotid Wallstent 7/40 mm (Boston Scientific, USA), and post dilation with a 5.5/20 mm semi-compliant balloon. A trivial plaque prolapse was shown at the final angiography, confirmed by an OCT evaluation (Figure 3). No further treatment was required. The patient was discharged two days after procedure without complications. The plaque prolapse was not present anymore at a six-month duplex scan follow-up.

**(4)**  **Cerebral hyperperfusion syndrome (CHS)**

CHS is a rare but severe carotid revascularization complication. A recent systematic review and meta-analysis reported that the incidence of CHS after CAS was between 3.1 and 6.8% [21]. CHS can be complicated by intracerebral haemorrhage (ICH) in 0.74% of cases [22]. Cerebral circulation autoregulation is one of the mechanisms contributing to the development of CHS. CHS risk factors are as follows: hypertension at baseline, treated carotid stenosis of >90%, a poor collateral blood flow defined by contralateral carotid occlusion or stenosis > 80%, and an isolated ipsilateral carotid circulation [23]. Among clinical and angiographic clues, trans-cranial Doppler (TCD) was shown to be an effective non-invasive test to stratify the CHS risk. Indeed, both ipsilateral and contralateral peak systolic velocity ratio > 2.4 (PSVR) measured in the middle cerebral artery, before and after carotid stenting, are independent CRI risk factors of CHS [24]. Patients with these risk factors may require more intensive hemodynamic monitoring after CAS, including prolongation of hospital stay. The clinical presentations of CHS include severe headache (ipsilateral to the lesion side or diffuse) and eye and facial pain. It is commonly associated with an increase in blood pressure. More severe symptoms like focal neurological deficits, seizures, and loss of consciousness are less common. Usually, patients develop symptoms during the first hours after carotid revascularization even if some late-onset symptoms have been described [22]. The instrumental diagnosis is basically based on CT scans or MRI. Patchy or diffuse white matter oedema, predominantly involving the posterior parieto–occipital lobe, focal infarction, and petechial haemorrhage are the most frequent findings [25,26]. A consensus on definition and diagnostic criteria for CHS using different hemodynamic assessment tools is warranted.

**Brief case description**

**Figure 4:**
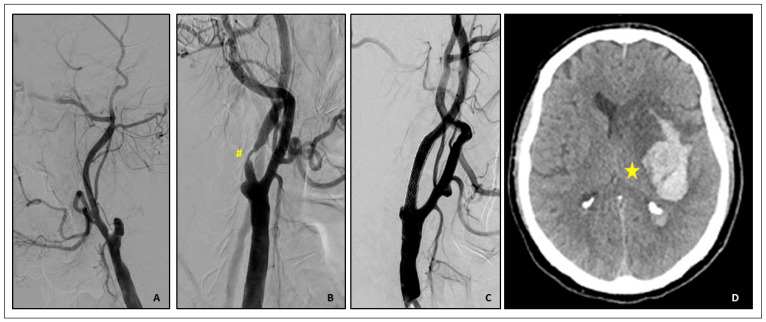
(**A**–**C**) Digital subtraction angiography (DSA). (**A**). Right internal carotid artery occlusion. (**B**). Tight left ICA stenosis (yellow blanket). (**C**). Final result after Carotid WallStent 9.0/30 mm (Boston Scientific, USA) deployment and post dilatation. (**D**) The brain CT scan, performed 2 h following carotid revascularization, shows a large right basal ganglia haemorrhage (star).

A 77-year-old man, with a previous right hemispheric stroke due to acute right internal carotid artery occlusion, complained of TIA during a hypertensive syndrome. The carotid duplex scan confirmed the previous right carotid artery occlusion and showed concomitant left severe internal carotid artery stenosis. The patient was scheduled for CAS. The procedural setting was as follows: distal cerebral protection by Filter EZ (Boston Scientific, USA), direct stenting (Carotid Wallstent 9/30 mm, Boston Scientific, USA) and post dilation with a 5.5/20 mm semi-compliant balloon. Two hours after CAS, the patient complained of a headache and manifested neurological hemispheric symptoms. Blood pressure was persistently high. Brain CT scan showed a large right basal ganglia haemorrhage. The patient was referred to the stroke unit and was discharged 14 days after the index procedure with a clinical major stroke pattern (Figure 4).

**(5)**  **Vascular access site complications**

Use of the radial artery in percutaneous coronary interventions (PCIs) was shown to be safer than femoral access in different randomized trials and meta-analyses; in fact, vascular access site complications such as pseudo-aneurysms, retroperitoneal haemorrhages, or groin haematomas can be avoided using a radial artery approach [27]. In the recent literature, very few data regarding the use of an RA approach in peripheral PTA are available, even if it is gaining increasing popularity, especially among interventional cardiologist operators. Some issues limiting the widespread use of the radial approach in peripheral procedures are radial artery diameter, the availability of dedicated devices, and a long learning curve to reach adequate expertise. A very recent meta-analysis showed that radial CAS can be performed with very high procedural success rates, around 90%; furthermore, among TRA complications, radial artery occlusion and forearm hematoma have been reported, respectively, in 5.9% and in 1.4% of cases [28]. Hereafter, we report a catastrophic complication that to our best knowledge has not yet been described in CAS procedures: radial artery long sheath entrapment requiring surgical intervention for sheath removal.

**Brief Complication description:**

**Figure 5:**
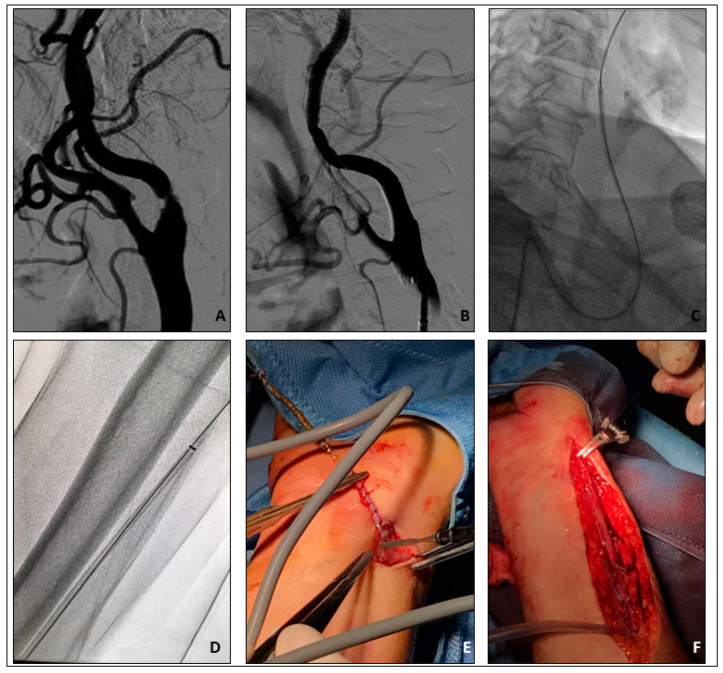
(**A**,**B**) DSA. (**A**). Very tight left ICA stenosis. (**B**). Final result after carotid stenting (Cristallo Ideale 7–10/40 mm). (**C**) 5 French Destination sheath pull back through the tortuous carotid–subclavian–axillar axis. (**D**) Destination sheath entrapment at the level of proximal right forearm. (**E**,**F**) Surgical sheath removal and radial artery surgical reconstruction.

A 74-year-old lady, complaining of a recent minor stroke due to severe left internal carotid artery stenosis, was scheduled for CAS because she was judged to be at high risk for carotid endarterectomy. The procedure was performed through the right radial artery, using a 5F 90 cm long sheath (Destination, Terumo, Tokyo, Japan) in a conventional fashion: embolic cerebral protection with a FilterWire EZ™ (Boston Scientific, USA); undersized balloon pre-dilatation with a semi-compliant balloon Emerge 3.0/20 mm (Boston Scientific, Massachusetts, USA); stenting with a Cristallo Ideale stent (Medtronic, Inc. USA); and post dilatation with a 5.5/20 mm semi-compliant balloon. The angiographic result was very satisfactory, but a lot of friction was felt during the long sheath removal. Indeed, the sheath tip was entrapped at the level of the right omeral artery due to severe and persistent arterial spasm. Any attempts to retrieve the sheath failed, although intra-arterial injection of nitrates or calcium-channel antagonists. Finally, the sheath was almost broken close to the haemostatic valve because of the vigorous attempts to pull back the sheath. A surgical brachial and radial artery arteriotomy was required to remove the Terumo Destination. The sheath wall was completely unravelled with the stainless-steel braided wire and separated by the plastic polymer (Figure 5).

## 4. Conclusions

Periprocedural stroke and vascular-access-site-related complications, even in cases of CAS performed through the radial artery route, are still the Achilles’ heel of this endovascular procedure. These complications can occur infrequently, even under the expert hands of experienced practitioners, potentially resulting in periprocedural strokes and extended hospital stays. Being aware of these complications, along with their management, can equip interventionalists with the necessary knowledge to further enhance their CAS performance.

## Figures and Tables

**Table 1:** Clinical and procedural characteristics.

Population: N = 1012	N%
Age (years)	72 ± 8.9
Men	739 (73)
Octogenarians	233 (23)
Hypertension	789 (78)
Hyperlipemia	749 (74)
Diabetes mellitus	575 (31.8)
Current or former smokers	993 (54.9)
Symptomatic	232 (23)
Asymptomatic	780 (77)
Coronary artery disease	210 (21)
Peripheral arterial disease	187 (18.5)
Vascular Characteristics	
Left ICA lesions	537 (53)
Right ICA lesions	475 (47)
% Stenosis	83±11
Contralateral disease	956 (52.9)
>70%	101 (10)
Total occlusion	30 (3)
Procedural Characteristics	
Technical success	1007 (99.5)
Radial access	132 (13)
Use of EPD	1012 (100)
Proximal protection	213 (21)
Distal protection	799 (79)
Pre-dilatation	213 (21)
Post-dilatation	951 (94)
Stent Type	
Open cells	81 (8)
Hybrid stent	192 (19)
Closed cells	638 (63)
Double layer	113 (11)

EPD = embolic protection device; ICA = internal carotid artery.

**Table 2:** Procedural and in-hospital complications.

Population: N = 1012	N%
Procedural success	967 (95.5)
MACCEs	
Total number	64 (6.3)
Stroke	35 (3.4)
Minor stroke	22 (2.1)
Major stroke	13 (1.3)
TIA	20 (1.9)
Death	6 (0.6)
Myocardial infarction	1 (0.3)
Vascular Complications	
Total number	54 (5.3)
Major hematoma	24 (2.3)
Minor hematoma	12 (1.1)
Retroperitoneal hematoma	3 (0.3)
Pseudoaneurysm	9 (0.9)
Arteriovenous fistula	4 (0.4)
Requiring surgery	4 (0.4)
Requiring blood transfusion	8 (0.8)
Others	2 (0.2)

MI = myocardial infarction; MACCE = major adverse cardiac and cerebrovascular events; TIA = transient ischemic attack.

## Data Availability

The data presented in this study are available on request from the corresponding author.
